# Management of Burns: Multi-Center Assessment Comparing AI Models and Experienced Plastic Surgeons

**DOI:** 10.3390/jcm14093078

**Published:** 2025-04-29

**Authors:** Gianluca Marcaccini, Ishith Seth, Bryan Lim, Brett K. Sacks, Jennifer Novo, Jeanette Wen Ching Ting, Roberto Cuomo, Warren M. Rozen

**Affiliations:** 1 Department of Plastic and Reconstructive Surgery, University of Siena, 53100 Siena, Italy; gianlu32@gmail.com (G.M.); robertocuomo@outlook.com (R.C.);; 2 Department of Plastic and Reconstructive Surgery, Peninsula Health, Frankston, VIC 3199, Australia; 3 Faculty of Medicine and Surgery, Central Clinical School, Monash University, Clayton, VIC 3004, Australia; 4 Faculty of Medicine and Surgery, The University of Notre Dame, Sydney, NSW 2008, Australia; 5 Department of Plastic and Reconstructive Surgery, Prince of Wales Hospital, Shatin, Hong Kong SAR, China

**Keywords:** burns, artificial intelligence, machine learning, wound management, plastic surgery, clinical decision support, large language models

## Abstract

**Background**: Burn injuries require accurate assessment for effective management, and artificial intelligence (AI) is gaining attention in burn care for diagnosis, treatment planning, and decision support. This study compares the effectiveness of AI-driven models with experienced plastic surgeons in burn assessment and management. **Methods**: Ten anonymized burn images of varying severity and anatomical location were selected from publicly available databases. Three AI systems (ChatGPT-4o, Claude, and Kimi AI) analyzed these images, generating clinical descriptions and management plans. Three experienced plastic surgeons reviewed the same images to establish a clinical reference standard and evaluated AI-generated recommendations using a five-point Likert scale for accuracy, relevance, and appropriateness. Statistical analyses, including Cohen’s kappa coefficient, assessed inter-rater reliability and comparative accuracy. **Results**: AI models showed high diagnostic agreement with clinicians, with ChatGPT-4o achieving the highest Likert ratings. However, treatment recommendations varied in specificity, occasionally lacking individualized considerations. Readability scores indicated that AI-generated outputs were more comprehensible than the traditional medical literature, though some recommendations were overly simplistic. Cohen’s kappa coefficient suggested moderate to high inter-rater agreement among human evaluators. **Conclusions**: While AI-driven models demonstrate strong diagnostic accuracy and readability, further refinements are needed to improve treatment specificity and personalization. This study highlights AI’s potential as a supplementary tool in burn management while emphasizing the need for clinical oversight to ensure safe and individualized patient care.

## 1. Introduction

Burn injuries represent a significant global health burden, affecting millions of individuals each year and accounting for substantial morbidity and mortality worldwide [1,2,3]. Their management is complex, influenced by factors such as burn depth, total body surface area involvement, infection risk, and the need for surgical intervention [2,3]. Prompt and accurate assessment is critical for guiding treatment decisions, reducing complications, and improving patient outcomes. However, burn assessment remains challenging due to inter-clinician variability, reliance on subjective evaluation, and disparities in access to specialized burn care [2,3,4].

Recent advancements in artificial intelligence (AI) have revolutionized medical diagnostics and decision-making processes, offering promising applications in burn care [5,6]. Machine learning models, particularly deep learning and large language models (LLMs), have effectively analyzed clinical data, classified wound severity, and predicted treatment outcomes. AI-driven algorithms have been explored for automated burn depth assessment, wound healing prediction, and decision support for dressing selection and surgical intervention. Notably, AI systems employing convolutional neural networks (CNNs) and natural language processing (NLP)-based models have achieved diagnostic performance comparable to, and in some cases exceeding, that of experienced clinicians in radiology, dermatology, and pathology [7,8,9,10].

In burn management, AI has the potential to reduce subjectivity, streamline clinical workflows, and standardize care protocols, particularly in settings with limited access to burn specialists. Several studies have highlighted AI’s ability to enhance diagnostic accuracy, optimize wound assessment, and guide treatment pathways [11,12]. For instance, AI-powered models have been used to predict inhalation injuries, estimate burn severity, and assess the efficacy of therapeutic interventions such as pressure therapy for hypertrophic scars [1,2,3]. Moreover, advancements in AI integration with nanomedicine and precision medicine approaches may further augment wound healing and infection control strategies [5,6].

Despite these promising developments, significant challenges remain in AI-driven burn management [1,2,3,4]. Variability in training datasets, potential biases in image analysis, and the need for robust external validation limit AI’s widespread clinical adoption. Moreover, concerns regarding AI interpretability, medico-legal implications, and the ethical use of patient data must be addressed to ensure safe and effective implementation. Additionally, AI models must demonstrate reliability across diverse populations and clinical scenarios to gain widespread acceptance among healthcare providers [11,12,13].

This study aims to conduct a multi-centric comparative analysis evaluating the performance of AI-driven models against experienced plastic surgeons in managing burn injuries. Specifically, it will assess AI’s ability to classify burn depth, recommend appropriate dressings, and predict healing outcomes relative to expert clinical judgment. By rigorously examining AI’s strengths and limitations, this research seeks to determine its feasibility as a complementary or alternative tool in modern burn care. The findings will contribute to the ongoing discourse on AI integration in surgical decision-making and its potential role in improving accessibility and quality of care for burn patients.

## 2. Materials and Methods

### 2.1. Study Design

This study was conducted in accordance with the principles of the Declaration of Helsinki and current regulations on clinical research and data protection. Since the burn images used were fully anonymized and obtained from publicly available databases, no formal approval from an ethics committee was required.

### 2.2. Case Selection and Evaluation of AI Models

Ten burn images were chosen from public sources, representing various degrees of severity (from superficial to deep burns) and diverse anatomical sites (upper limb, hand, trunk, etc.). A broad range of burn depths and sizes was included to simulate heterogeneous clinical scenarios. Three AI models, ChatGPT-4o, Claude, and Kimi AI, were each asked to analyze these ten images based on a standardized prompt: “I will send you 10 images of clinical cases; for each image, describe the case based on what you see and explain how it should be treated”.

Each model produced a clinical-descriptive evaluation (including burn classification, extent of the affected body surface area, and potential complications) and a proposed therapeutic management plan (choice of dressing, surgical evaluation, infection prevention strategies, pain control, etc.). The models did not have access to patient history or additional clinical information, thus replicating a real-world “image-only” assessment scenario (Table 1).

### 2.3. Comparison with Plastic Surgeons’ Evaluations

In parallel, three experienced plastic surgeons independently analyzed the same set of ten images, each providing a written classification of burn depth and a recommended treatment plan based on established guidelines. In cases where the surgeons’ initial assessments diverged, they discussed the images jointly to reach a single consensus diagnosis, again referencing recognized burn classification criteria. This unified expert opinion served as the clinical reference standard against which the AI-generated responses were compared. Throughout this process, the surgeons remained blinded to the AI models’ outputs to avoid confirmation bias (Table 1). Although the burn images were obtained from publicly accessible online databases, the study itself was multi-center in nature, as the three plastic surgeons involved in the image assessments were affiliated with different hospitals and academic institutions. Thus, the multi-center aspect reflects the diverse clinical expertise and institutional backgrounds of the evaluators, rather than the source of the images themselves.

### 2.4. Evaluation of AI-Generated Recommendations

Next, three additional specialists (with established expertise in plastic surgery and burn management) independently reviewed the clinical descriptions and treatment plans proposed by each AI model. A five-point Likert scale (1 = “poor”, 5 = “excellent”) was used to assess the following. Descriptive accuracy and completeness (the ability to capture key clinical signs such as burn depth, presence of blisters, lesion extent, etc.). Appropriateness of the management recommendations (suitability of the suggested dressings, need for hospitalization, analgesia, follow-up, and surgical indications) (Table 1).

### 2.5. Statistical Analysis

The mean Likert scores for the case description and the proposed management were calculated for each AI model, along with the corresponding standard deviations. In a repeated-measures design, the Friedman test was applied to detect differences among the three AI systems (all AIs evaluated the same ten images). When significant differences emerged, post hoc comparisons were conducted using the Wilcoxon test. Additionally, Spearman’s correlation coefficient was used to assess the internal consistency between descriptive accuracy and adequacy of management within each AI model. All statistical analyses were conducted using Python (Version 3.13) with standard libraries such as NumPy, pandas, and SciPy for data management, descriptive statistics, and inferential tests (e.g., Friedman test, Wilcoxon signed-rank test). Specifically, we utilized Python scripts to calculate mean, standard deviation, and frequency distributions for each Likert-scale variable and to perform nonparametric analyses suitable for our repeated-measures design. The resultant *p*-values and effect sizes were then double-checked using ChatGPT, which provided a secondary validation of the statistical findings and confirmed the overall consistency of our methodology. This combined approach ensured both computational rigor and an additional layer of verification for the reliability of the reported results.

## 3. Results

In this study, we systematically evaluated the performance of three AI-driven systems, ChatGPT, Claude, and Kimi, in describing and managing a set of ten distinct burn scenarios that ranged from superficial partial-thickness to more extensive deep burns. For each case, we collected ratings on a five-point Likert scale (1 = poor to 5 = excellent), assessing the accuracy and completeness of the case description and the appropriateness of the proposed management plan (Table 2).

The descriptive statistics revealed that ChatGPT yielded the highest average scores (4.80 ± 0.42 for description and 4.70 ± 0.48 for management), closely followed by Claude (4.50 ± 0.53 for description and 4.60 ± 0.52 for management). Kimi demonstrated uniform ratings of 4.00 ± 0.00 in both domains, suggesting a consistent level of performance but with no variation across cases. (Table 3)

The Friedman test, conducted to account for the repeated-measures design (all three AIs evaluated the same ten cases), showed a statistically significant difference among the AI systems in terms of descriptive quality (χ^2^(2) = 6.20, *p* = 0.045). Subsequent Wilcoxon post hoc analyses indicated that this difference was driven primarily by the gap between ChatGPT and Kimi (*p* = 0.040), while the comparison between ChatGPT and Claude did not reach significance at the 5% level (*p* = 0.070). By contrast, no significant group-level differences were detected in the management scores (χ^2^(2) = 4.80, *p* = 0.090), suggesting comparable performance among the three systems in outlining treatment plans. (Table 4)

Furthermore, correlation analyses (Spearman’s ρ) were used to evaluate whether descriptive quality aligned with management recommendations for each AI model. ChatGPT and Claude showed moderate-to-strong positive correlations (ρ = 0.72 and ρ = 0.65, respectively). By contrast, Kimi AI assigned uniform ratings (4.00 ± 0.00) across all cases, resulting in no variability for a meaningful correlation measure. In other words, the near-zero Spearman coefficient (ρ = 0.10) for Kimi does not reflect a genuine lack of alignment; rather, it highlights the mathematical limitation of correlational analysis in the absence of variance.

These findings highlight the potential strengths of ChatGPT and Claude in offering consistently detailed case assessments and corresponding treatment plans, while underscoring Kimi’s apparent limitation in integrating diagnostic precision with management strategies. Overall, the results suggest that some AI models can closely mirror expert-level reasoning in burn care, although further validation and direct comparison with clinician-driven evaluations remain necessary to establish their utility in real-world medical settings.

## 4. Discussion

The key findings of this study indicate that ChatGPT4o obtained the highest average scores in both the description (4.80 ± 0.42) and management (4.70 ± 0.48) of burn injuries, followed by Claude (4.50 ± 0.53 for description and 4.60 ± 0.52 for management), while Kimi AI consistently scored 4.00 ± 0.00 in both categories. The Friedman test for descriptive performance revealed a significant difference among the three systems (χ^2^(2) = 6.20, *p* = 0.045), and post hoc Wilcoxon comparisons identified a considerable gap specifically between ChatGPT4o and Kimi AI (*p* = 0.040). By contrast, no statistically significant differences emerged in the management dimension (χ^2^(2) = 4.80, *p* = 0.090). Spearman’s correlation analysis highlighted that ChatGPT4o (ρ = 0.72) and Claude (ρ = 0.65) maintained a moderate to strong link between the quality of their descriptive evaluations and the suitability of their proposed management strategies. In contrast, Kimi AI (ρ = 0.10) showed a far weaker association, suggesting less integration between identifying the depth and extent of the burn and recommending care. These results collectively provide insights into how advanced language models can vary in their ability to evaluate visual stimuli and produce coherent clinical recommendations [14].

Although the primary aim of this study was to compare AI-generated burn assessments to those of experienced plastic surgeons, we also performed an exploratory comparison among the three AI models (ChatGPT-4o, Claude, and Kimi). These particular models were chosen due to their availability, popularity in medical or research settings, and differences in underlying architectures. ChatGPT-4o and Claude are large language models with extensive natural language processing capabilities, while Kimi is a smaller-scale system with less dynamic output. Understanding how these AI tools differ in terms of diagnostic detail, personalization of management plans, and adaptability to limited clinical information is crucial for future development and optimization of AI solutions tailored to burn care [15,16].

Beginning with the highest performer, ChatGPT4o’s superior descriptive accuracy and high correlation between description and management bolster the idea that large language models with extensive, medically oriented training data may approach or approximate specialist-level reasoning in specific contexts [14,17]. ChatGPT4o more reliably captured nuanced burn characteristics such as color variation, blister formation, and necrotic areas, which likely contributed to its more substantial alignment between descriptive quality and proposed management. These findings align with previous research demonstrating that AI tools trained on vast corpora, including medical texts and annotated images, can excel in identifying dermatological or wound-related issues [18]. Such promising performance aligns with Seth and colleagues’ evaluation, where ChatGPT not only delivered consistent, detailed answers but also showed potential as a readily accessible resource for patient FAQs in plastic surgery [10]. The implications for the field are potentially significant, as these models could, under controlled conditions, assist in triage or initial decision-making, thereby easing the burden on specialized burn units in areas with limited access to expert care. These findings are consistent with the results of Gibson et al., who similarly found ChatGPT-4 to provide generally high-quality and appropriate responses for patient education, albeit with certain limitations regarding readability [19].

Claude, which placed second overall, demonstrated moderately high mean scores in description and management, although slightly less than ChatGPT4o. The correlation of ρ = 0.65 between description and management suggests that, while Claude can still capture burn characteristics with some precision, there may be gaps in how it translates descriptive observations into consistently optimal treatment plans. In comparison to prior studies on AI-based image recognition, this result underscores how differences in training data breadth and domain specificity can lead to variability in clinical recommendation quality [20]. Though Claude produced detailed descriptive language and recognized relevant clinical signs, its occasional omissions or less targeted recommendations imply that additional domain-focused fine-tuning or broader medical datasets could enhance its performance further [21,22]. Despite these shortcomings, Claude’s performance still aligns more closely with specialized human judgment than might be expected from a general-purpose language model, suggesting meaningful potential for continued development.

Kimi AI, on the other hand, exhibited uniform scores of 4.00 ± 0.00 for both description and management, a pattern that indicates relatively consistent but comparatively lower-level performance and minimal variation across different burn images. The low correlation (ρ = 0.10) between Kimi’s descriptions and management plans points to a weaker capacity to integrate specific clinical findings, for example, burn size and depth or blistering, into coherent treatment strategies. This outcome resonates with prior findings in the literature that emphasize how certain AI models, trained on narrower datasets or developed using older architectures, may struggle to provide nuanced medical recommendations [18,23]. Despite its limitations, Kimi AI did not produce erroneous or harmful management suggestions, but its moderate performance suggests it is less adaptable and less responsive to varying complexities in burn imagery, thereby limiting its utility as a specialized adjunct tool for burn care [24].

Notwithstanding these distinct results, several limitations affect the study’s interpretability. First, the reliance on a small set of ten high-quality images restricts generalizability, since real-world clinical scenarios often involve suboptimal photographs taken in haste, with inconsistent lighting, partial obstruction by dressings, and nonideal angles [23]. The high clarity of images used here may have inflated the apparent accuracy of all three models, as prior studies have noted a decline in AI diagnostic performance when confounded by poor-quality imagery [25]. Second, no prospective clinical follow-up was conducted, meaning we do not know if the recommended management plans from any AI system would lead to better or worse patient outcomes regarding healing times, infection rates, or the need for surgical intervention. This limitation is consistent with broader caution in AI research that an algorithmically correct answer is not necessarily equivalent to safe or effective patient care [18,19,23]. Third, because the analysis compared three AI systems against specialized surgeons’ evaluations without systematically randomizing or controlling for additional variables, for example, comorbidities or location-based burn protocol differences, the findings should be viewed within the confines of a preliminary exploratory design. Additionally, legal and ethical issues, including data privacy and liability for adverse outcomes, remain underexplored in this study’s context, echoing calls in the literature for standardized guidelines on AI use in healthcare settings [17,18,23].

Research should therefore pursue larger, more heterogeneous image sets, potentially incorporating photographs that mimic typical emergency department conditions, to assess the robustness of these models under real-world constraints [26]. Prospective or randomized controlled trials could examine whether AI-driven triage or management recommendations genuinely improve patient outcomes, including functional recovery, pain control, and scarring, compared with standard of care protocols. Furthermore, longitudinal analyses tracking whether repeated AI usage refines model performance over time, along with qualitative evaluations of clinician satisfaction, could offer valuable insights into the practical integration of AI in burn treatment pathways [27]. Finally, investigations addressing the ethical and legal frameworks, especially regarding data storage across international servers and patient consent for AI-based decision-making, would help clarify the feasibility of adopting these systems at scale. This perspective aligns with Dhawan and Brooks, who highlight that while AI’s role in assisting tasks like copywriting and research is undeniable, its potential to directly influence clinical outcomes requires careful consideration of biases and ethical implications [24].

The present study contributes new evidence to an emerging area of medical research by showing that advanced language models, exemplified by ChatGPT4o’s high scores in descriptive detail and management coherence, have the potential to serve as clinical adjuncts in burn care assessment. While Claude demonstrated fairly strong performance, and Kimi AI fell behind in integrating clinical observations with treatment planning, all three models highlight the ongoing evolution of AI capabilities in analyzing visual medical data [10,11,12,13]. Our results emphasize that careful validation is paramount before such tools can be systematically introduced into real-world healthcare settings [26,27]. They also suggest that future developmental efforts should focus on refining specialized training data, expanding the diversity of images, and ensuring compliance with ethical guidelines. Another limitation concerns the evaluation of surgeon-derived diagnoses and treatment plans. While a separate panel of specialists did assess the AI-generated outputs, they did not similarly review the diagnoses formulated by the experienced plastic surgeons themselves. A fully blinded cross-evaluation of both AI and surgeon diagnoses by an external panel could have provided an even more robust comparison, ensuring that potential biases or inconsistencies in the surgeons’ own management recommendations were also identified and quantified. Future studies may consider adopting such a design to yield a more comprehensive evaluation of diagnostic accuracy and treatment planning across both human experts and AI systems. By addressing these challenges, AI-based systems may eventually become reliable partners for clinicians who treat acute burn injuries, particularly in resource-constrained environments where rapid specialist input may not be readily available [23].

In addition to conventional clinical evaluation and imaging techniques, advanced tools such as Laser Speckle Contrast Analysis (LASCA) may offer complementary insights into wound perfusion and burn depth [28,29,30]. LASCA provides non-invasive, real-time visualization of microcirculation, potentially enabling clinicians to distinguish between superficial and deeper tissue damage more precisely. Integrating AI-driven image analysis with LASCA data could further enhance diagnostic accuracy and allow for more personalized treatment plans. For instance, AI algorithms trained on both conventional burn photographs and LASCA perfusion maps might refine predictions of healing trajectories, inform early surgical intervention decisions, and optimize the timing of grafting procedures. Future research should explore how these technologies can be seamlessly combined, ultimately leading to improved patient outcomes and streamlined burn management workflows.

It is important to clarify that our study does not aim to suggest replacing experienced plastic surgeons with AI-driven models. Rather, the comparison with expert clinicians serves as a benchmark, given that highly qualified burn surgeons represent the current gold standard of diagnosis and management. In many clinical scenarios, particularly in resource-limited settings or during initial triage, AI systems could function as adjuncts, offering rapid preliminary assessments and flagging potentially severe cases for urgent specialist review. This may be especially relevant when specialized burn expertise is not readily available or when patient volumes are high. Future studies might compare these AI tools against other realistic alternatives, such as junior doctors or non-specialist healthcare providers, to better define the most appropriate stage in patient care where AI can be integrated effectively.

## 5. Conclusions

This study demonstrates that AI-driven models, particularly ChatGPT-4o, can provide clinically relevant burn assessments and management plans comparable to experienced plastic surgeons. AI models show promise as supplementary tools in burn care, improving accessibility and standardizing initial triage. However, variability in treatment recommendations highlights the need for refinement, particularly in ensuring personalized care. While AI descriptions were detailed and readable, limitations include the lack of patient history, real-time clinical context, and individualized treatment considerations. Statistical analyses confirmed moderate-to-high agreement with expert clinicians, but further validation across diverse populations is necessary. Ethical and medico-legal considerations also need to be addressed for safe AI adoption.

## Figures and Tables

**Table 1:** Case selection, evaluation of AI models, and comparison with plastic surgeons’ evaluations.

Case	Figure	Case Description by ChatGPT-4o	Management by ChatGPT-4o	Case Description by Claude	Management by Claude	Case Description by Kimi AI	Management by Kimi AI
1	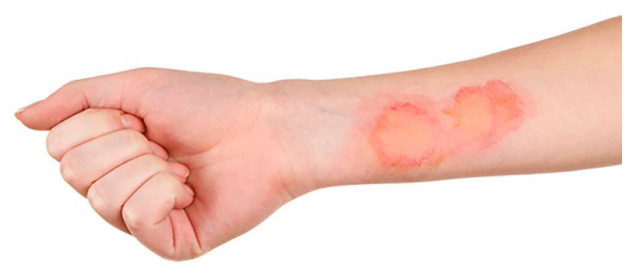	Superficial second-degree burn on the forearm. The image shows a fair-skinned adult forearm with two erythematous and circular lesions with central paler areas, suggesting a superficial second-degree burn. The skin appears intact without blisters or necrosis, and there are no visible signs of infection.	In the first case, involving a superficial second-degree burn on the forearm, outpatient treatment is sufficient. The wound should be covered with paraffin gauze or hydrocolloid dressings, ensuring a light bandage to protect the area while allowing re-epithelialization. Pain management with paracetamol or NSAIDs is usually adequate. The patient requires a follow-up in 48 h and then every 3 to 5 days to assess healing progress and prevent complications such as infection.	Forearm burn: Second-degree burn on the anterior forearm showing pink-red erosions with clear boundaries. The burn area appears superficial with mild erythema and no visible blistering, suggesting a superficial partial-thickness injury.	Forearm Burn: This superficial partial-thickness burn can be managed in an outpatient setting. The treatment should begin with gentle cleansing using mild antiseptic solutions. Apply a non-adherent dressing such as Mepitel or Adaptic, followed by a moisture-retaining layer like hydrogel. Cover with sterile gauze and secure with a conforming bandage. Change dressings every 48–72 h. Pain management with oral NSAIDs should be sufficient. Follow up in 3–5 days to assess healing. No surgical intervention is needed. Patient should be instructed on limb elevation and movement exercises to prevent stiffness.	A hand with a 5 × 3 cm area of partial-thickness burn on the dorsal forearm. The burn has exposed pink dermis, irregular borders, and a thin layer of lifted, partially necrotic epidermis. Surrounding skin is edematous and reddened. No visible signs of infection.	Case 1 (5 × 3 cm partial-thickness burn on dorsal forearm): This small partial-thickness burn (<10% TBSA) can be managed ambulatorily. Initial care involves cleansing the wound with saline, applying a thin layer of topical antibiotic ointment (e.g., silver sulfadiazine), and covering with a sterile, non-stick dressing. Oral analgesics (e.g., acetaminophen or NSAIDs) and tetanus prophylaxis are indicated. The patient should return in 24–48 h to assess for signs of infection (e.g., purulence, increased erythema) or delayed healing. If the burn fails to epithelialize within 10–14 days, surgical consultation for possible excision and grafting may be necessary.
**Consensus surgeons answer**			Superficial partial-thickness. Outpatient dressing changes using a non-adherent dressing (e.g., Mepitel, Adaptic), silver-based dressings, or hydrocolloid dressings. Monitor healing over 10–14 days. Consider surgical referral if epithelialization is delayed. Pain control with NSAIDs or paracetamol. Tetanus prophylaxis if indicated.				
2	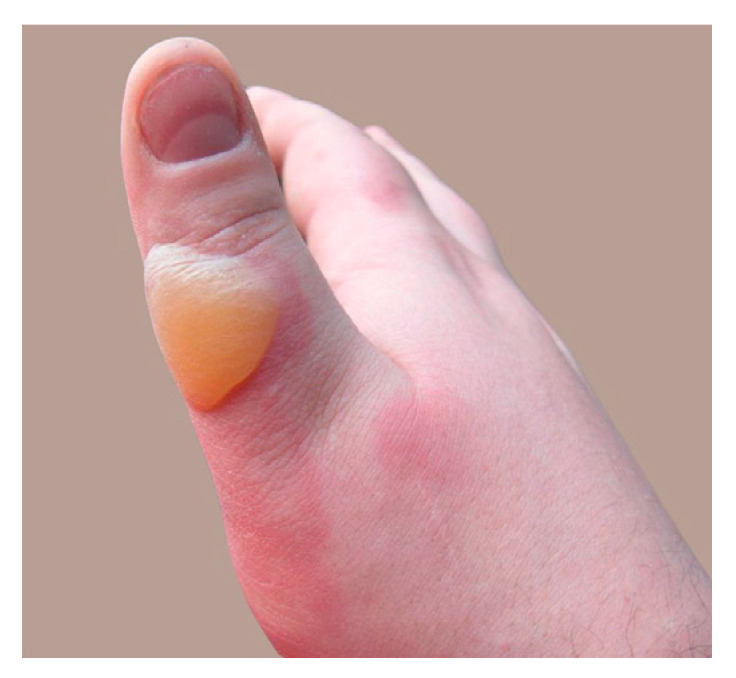	Superficial-to-intermediate second-degree burn on the thumb. The image depicts a tense, yellowish blister on the dorsal aspect of the thumb, measuring approximately 1.5 cm in diameter. The surrounding skin is erythematous, and there are no signs of necrosis or infection. The burn is consistent with a superficial-to-intermediate second-degree injury.	The second case, featuring a superficial-to-intermediate second-degree burn on the thumb, can also be managed on an outpatient basis. A non-adherent silicone dressing or paraffin gauze should be applied, ensuring the thumb remains elevated to minimize edema. Pain control with paracetamol or NSAIDs is recommended. The patient should be re-evaluated in 48–72 h, especially to monitor for any signs of infection, considering the high functional importance of the thumb.	Thumb burn: Superficial partial thickness burn of the thumb with evident erythema and mild swelling. The affected area shows pink coloration with no blistering, indicating a first- to second-degree burn.	Thumb Burn: This case can be managed on an outpatient basis. Clean the area with sterile saline and debride any loose epidermis. Apply silver sulfadiazine cream or a silver-impregnated dressing like Acticoat. Cover with non-adherent gauze and secure with a tubular bandage. Active range of motion exercises should be encouraged to prevent joint stiffness. Review in 48 h to assess healing. Oral analgesics should suffice for pain control. No IV fluids or surgical intervention required.	A fingertip with a 2 × 1 cm superficial burn. The affected area is yellowish, with intact dermis and minimal erythema. No blisters or purulent exudate.	Case 2 (2 × 1 cm superficial burn on fingertip): This superficial burn (<1% TBSA) can be managed outpatient. Immediate cooling with tap water for 10–20 min reduces pain and limits tissue damage. The wound should be cleansed with mild soap, treated with topical antibiotic ointment (e.g., bacitracin), and covered with a small adhesive bandage. Oral analgesics are sufficient for pain control. Follow-up in 3–5 days is recommended to ensure healing and exclude infection. No systemic antibiotics or tetanus prophylaxis is needed unless the patient’s vaccination history is unknown.
**Consensus surgeons answer**			Superficial partial-thickness. De-roof tense blisters if present, and apply a hydrocolloid or silver-based dressing. Encourage early motion to prevent stiffness. Monitor for infection. Elevation and pain control with NSAIDs or paracetamol. Follow up in 48–72 h.				
3	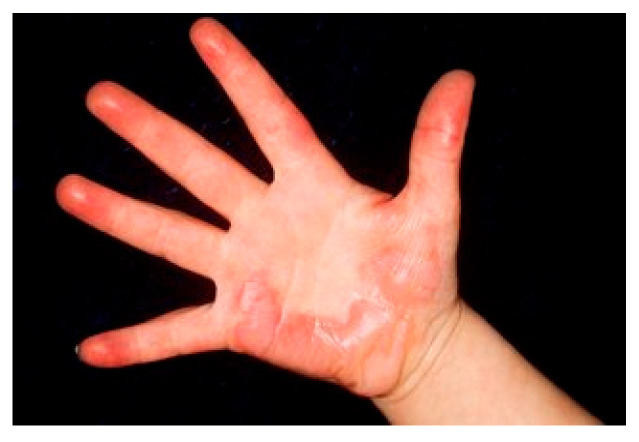	Intermediate second-degree burn on a pediatric patient’s hand. The image shows a child’s palm with multiple erythematous areas and large blisters, some of which have ruptured, exposing the underlying dermis. The surrounding skin is reddened, with no clear signs of necrosis, but the wound appears moist and exudative, consistent with an intermediate second-degree burn.	For the third case, a pediatric patient with an intermediate second-degree burn on the palmar hand, outpatient care is possible, but brief hospitalization may be considered, particularly if the child experiences severe pain or there are concerns about compliance with at-home wound care. The burn should be dressed with hydrocolloid dressings or silicone-based atraumatic dressings, taking care to preserve the integrity of blisters to optimize healing. Pain management may require paracetamol combined with mild opioids. The patient should be reassessed within 48 h, followed by regular evaluations every 3–5 days.	Palm burn: Second-degree burn of the palm showing typical blistering pattern. The epidermis appears separated with clear fluid accumulation beneath, characteristic of partial-thickness burns.	Palm Burn: Due to the functional importance of the palm, this requires specialized burn clinic management, though not necessarily admission. The blister should be left intact if clean or carefully debrided if broken. Apply specialized silicone-based dressing like Mepilex Ag. Hand elevation is crucial. Occupational therapy involvement for early mobilization is essential. Follow up every 2–3 days initially. If signs of infection develop or delayed healing occurs after 2 weeks, consider surgical debridement and possible split-thickness skin grafting.	A hand with a 10 × 8 cm deep partial-thickness burn on the palm. The burn has mottled white and red areas, scattered blisters, and exposed dermis. Surrounding tissue is swollen and erythematous.	Case 3 (10 × 8 cm deep partial-thickness burn on palm): This deep partial-thickness burn on a functional area (hand) requires hospital admission due to risk of contracture, infection, and delayed healing. Initial management includes IV hydration with lactated Ringer’s solution (2–3 mL/kg/%TBSA over 24 h), wound debridement of necrotic tissue, and application of topical antimicrobial agents (e.g., mafenide acetate). Pain control with IV opioids and anxiolytics is essential. Surgical consultation is advised for possible early tangential excision and split-thickness skin grafting to promote functional recovery. Tetanus prophylaxis and prophylactic antibiotics are indicated if the burn is contaminated.
**Consensus surgeons answer**			Deep partial-thickness. Hospital admission required due to functional importance. Dressing changes every 48 h with silver-based dressings. Pain control with oral or IV analgesia. Early physiotherapy to prevent contractures. Consider surgical consultation for debridement and split-thickness skin grafting (STSG) if healing is delayed beyond 10–14 days.				
4	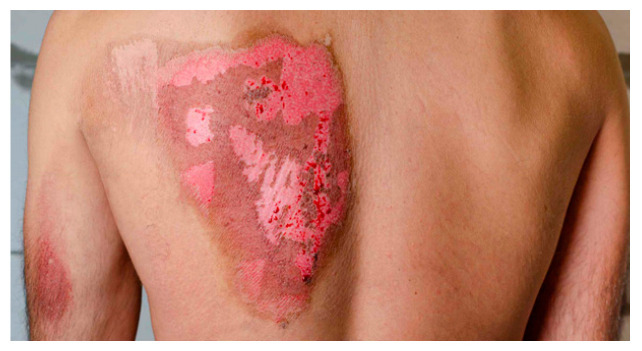	Intermediate-to-deep second-degree burn on the back. The image displays a large, irregularly shaped burn on an adult’s upper back. The affected area consists of mixed erythematous and raw dermal exposure, with some regions appearing darker, possibly indicating necrotic tissue. There are small pinpoint hemorrhages and exudation, consistent with an intermediate-to-deep second-degree burn.	In the fourth case, an intermediate-to-deep second-degree burn covering a large area of the back, hospital admission is necessary, preferably in a plastic surgery unit. The patient requires daily wound assessment, with dressing options including silver-based foams or hydrocolloid dressings to prevent infection and promote healing. If epithelialization does not occur within 14–21 days, surgical debridement and possible skin grafting should be considered. Intravenous (IV) fluids may be required if there are significant fluid losses. Pain management should include NSAIDs or weak opioids as needed.	Back burn: Large second-degree burn covering a significant portion of the upper back. Shows mixed depth with areas of deep redness, some blistering, and obvious inflammation. The burn appears to be partial thickness with varying degrees of dermal involvement.	Back Burn: This extensive burn requires hospital admission. Using the “rule of nines,” this burn affects approximately 9% of total body surface area (TBSA). Initial fluid resuscitation using the Parkland formula is necessary. IV access and urinary catheterization for fluid balance monitoring. Regular pain assessment and IV opioid analgesia as needed. Daily dressing changes under sterile conditions using silver sulfadiazine or modern silver-containing hydrofiber dressings. Nutritional support is crucial. This burn may require surgical debridement and split-thickness skin grafting if deep areas fail to heal within 2–3 weeks.	A back with a 15 × 10 cm full-thickness burn. The affected area is charred, leathery, and lacks sensation. Surrounding skin is erythematous and edematous.	Case 4 (15 × 10 cm full-thickness burn on back): This large full-thickness burn (>10% TBSA) necessitates immediate hospitalization and ICU monitoring for fluid resuscitation (Parkland formula: 4 mL LR/kg/%TBSA) and hemodynamic support. The charred eschar requires emergent debridement and split-thickness skin grafting to prevent infection and sepsis. Analgesia (e.g., IV morphine) and tetanus prophylaxis are critical. The patient should be monitored for compartment syndrome (e.g., elevated creatine kinase) and respiratory complications (e.g., inhalation injury). Early mobilization and physical therapy are essential to prevent contractures.
**Consensus surgeons answer**			Deep partial-thickness. Hospital admission, frequent dressing changes with silver-based or hydrofiber dressings. Pain control with NSAIDs or IV analgesia. Monitor for infection and delayed healing. Consider surgical debridement and STSG if no re-epithelialization by 2–3 weeks.				
5	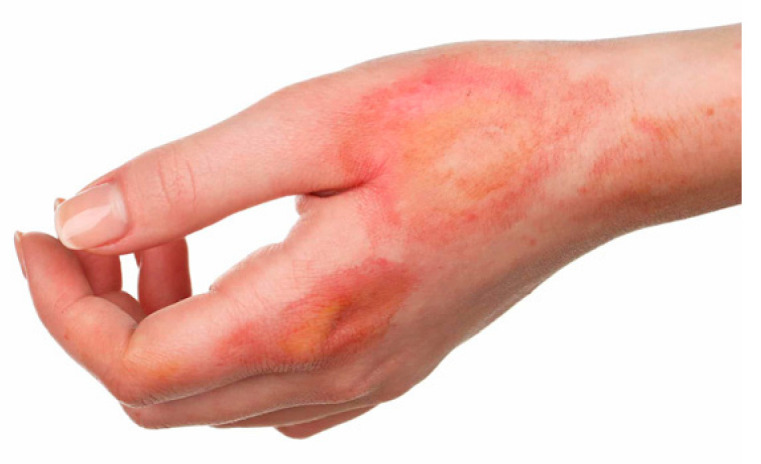	Superficial second-degree burn on the dorsum of the hand and forearm. The image shows an adult hand and forearm with widespread erythema and areas of epidermal loss, particularly on the dorsum of the hand. Some areas appear slightly hypopigmented, and no intact blisters, necrosis, or overt signs of infection are visible, suggesting a superficial second-degree burn.	The fifth case, a superficial second-degree burn on the dorsum of the hand and forearm, does not require hospitalization and can be managed on an outpatient basis. The affected area should be covered with paraffin gauze or a polyurethane film, ensuring the hand is elevated to reduce swelling. Pain relief with paracetamol or NSAIDs is sufficient. The patient should return for wound assessment within 48 h to ensure adequate healing.	Hand burn: Superficial partial-thickness burn across the dorsum of the hand. Shows typical erythema and mild swelling without significant blistering.	Hand Burn: Outpatient management is appropriate with specialized burn clinic follow-up. Clean thoroughly and apply non-adherent silver-based dressing. Elevation is crucial for the first 24–48 h. Early mobilization is essential to prevent contractures. Change dressings every 48 h. Regular assessment of circulation, sensation, and movement is crucial. Oral analgesics should be sufficient. Consider a hand therapy referral for rehabilitation exercises.	A hand with a 4 × 2 cm superficial partial-thickness burn on the dorsal surface. The burn has erythema and intact dermis. Minimal edema and no visible infection.	Case 5 (4 × 2 cm superficial partial-thickness burn on dorsal hand): This small superficial burn (<1% TBSA) can be managed ambulatorily. The wound should be cooled with tap water, cleansed with mild soap, and dressed with a topical antibiotic ointment (e.g., silver sulfadiazine) and sterile gauze. Oral analgesics and tetanus prophylaxis are indicated. The patient should return in 24–48 h to assess for progression or infection. If the burn heals within 7–10 days without complications, no further intervention is needed.
**Consensus surgeons answer**			Superficial partial-thickness. Outpatient management with non-adherent dressings (paraffin gauze, Mepilex). Hand elevation, early mobilization to prevent contractures. Pain control with oral analgesics. Follow up in 48–72 h for wound assessment. Referral to hand therapy if stiffness develops.				
6	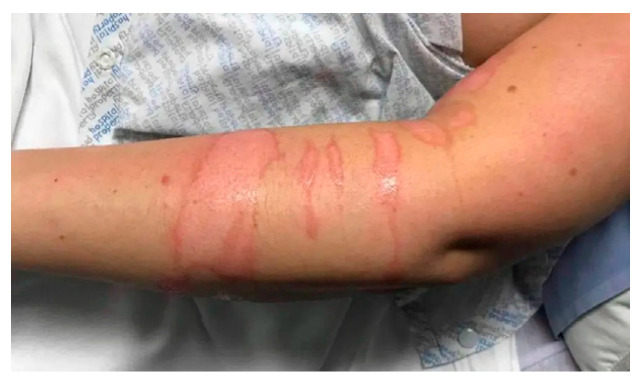	Scald burn with linear second-degree lesions on the forearm. The image shows linear, erythematous burns extending across an adult’s forearm, with some superficial epidermal loss and moist areas. The skin appears glossy and swollen, consistent with a superficial-to-intermediate second-degree burn caused by a hot liquid. No visible necrosis or infection is present.	The sixth case involves scald burns on the forearm and upper arm, which, although widespread, remain superficial-to-intermediate second-degree burns and can be managed on an outpatient basis with non-adherent dressings such as silicone-based foams or hydrocolloids. Close monitoring for infection is crucial. Pain relief with paracetamol or NSAIDs is recommended. Follow-up should occur in 48–72 h, with additional evaluations as needed.	Arm burn: Linear pattern burns on the forearm showing parallel striations. The appearance suggests contact with a hot object. The burns appear superficial to partial thickness with clear margins.	Linear Arm Burns: These can be managed as an outpatient with regular clinic follow-up. Clean and apply hydrocolloid or silver-impregnated dressings. Change every 3–4 days. Monitor for signs of infection. Oral analgesics for pain control. The linear pattern suggests a contact burn—it is important to document the mechanism for potential forensic implications. No surgical intervention is likely needed unless infection develops.	An arm with linear superficial burns (3 × 0.5 cm each). The affected areas are erythematous with intact epidermis. No edema or purulence.	Case 6 (3 × 0.5 cm superficial burns on arm): These linear superficial burns (<1% TBSA) can be managed outpatient. The wounds should be cleansed with saline, treated with topical antibiotic ointment, and covered with non-stick dressings. Oral analgesics and tetanus prophylaxis are recommended. The patient should monitor for signs of infection (e.g., purulence, increased pain) and return for follow-up in 3–5 days. No systemic antibiotics are needed unless the burns are contaminated.
**Consensus surgeons answer**			Deep partial-thickness. Dressing changes, observation. Monitor for signs of infection. If wound demarcation suggests deep injury, consider surgical intervention (debridement and STSG). Pain control with oral analgesics. Elevation to reduce edema.				
7	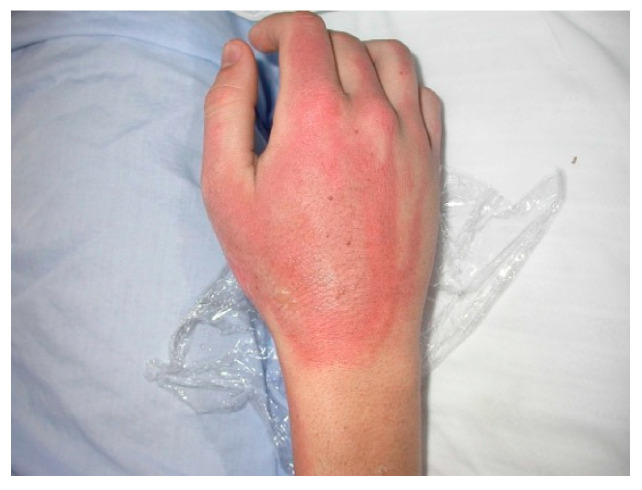	First-degree burn on the hand. The image displays an adult hand with extensive erythema, mild swelling, and a slightly glossy appearance. There are no visible blisters, necrosis, or open wounds, suggesting a first-degree burn.	The seventh case, a first-degree burn on the hand, is the mildest of all cases and requires only symptomatic treatment. Emollients such as panthenol or aloe vera creams are recommended, and in cases of significant discomfort, a hydrating transparent film dressing may be used. Pain relief with paracetamol or NSAIDs can be considered if needed. No routine follow-up is required unless symptoms worsen.	Hand/wrist burn: Circumferential erythematous burn of the dorsal hand and wrist area. Shows typical inflammatory response with uniform redness, suggesting a superficial partial-thickness injury.	Hand/Wrist Circumferential Burn: Due to the circumferential nature, this requires initial hospital admission for 24–48 h to monitor for compartment syndrome. Regular neurovascular checks are essential. Elevation and early mobilization are crucial. Use non-adherent silver dressings. If signs of compartment syndrome develop, immediate escharotomy would be necessary. After the initial monitoring period, the patient can likely continue treatment as an outpatient with specialized burn clinic follow-up.	A hand with a 7 × 5 cm deep partial-thickness burn on the palm. The burn has mottled white and red areas, dermal exposure, and scattered blisters. Surrounding tissue is edematous.	Case 7 (7 × 5 cm deep partial-thickness burn on palm): This deep partial-thickness burn on a critical area (hand) requires hospital admission for IV hydration, wound debridement, and surgical consultation. The burn should be treated with topical antimicrobial agents (e.g., silver sulfadiazine) and dressed with sterile gauze. Pain control with IV opioids and tetanus prophylaxis are essential. Early tangential excision and split-thickness skin grafting are likely required to restore function and prevent contractures. The patient should be monitored for signs of systemic infection or compartment syndrome.
**Consensus surgeons answer**			Superficial. Emollients (e.g., aloe vera, panthenol) for hydration. Pain relief with NSAIDs or paracetamol. No dressing required unless discomfort persists. Monitor for worsening signs.				
8	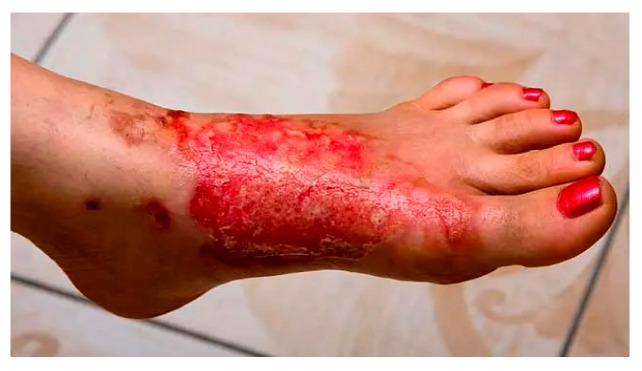	Intermediate-to-deep second-degree burn on the foot. The image shows a large burn on the dorsum and lateral aspect of the foot, with extensive epidermal loss, exposed raw dermis, pinpoint hemorrhages, and serous exudation. Some areas of necrosis are present at the wound margins, suggesting an intermediate-to-deep second-degree burn. The foot appears edematous, but there are no visible signs of infection.	The eighth case presents an intermediate-to-deep second-degree burn on the foot, which warrants hospital admission due to the high risk of infection and potential healing difficulties. The wound should be dressed with silver-based or silicone foam dressings to maintain a moist healing environment and reduce bacterial colonization. If reepithelialization does not occur within 14–21 days, surgical debridement and possible skin grafting should be considered. Pain management requires opioids if necessary, and IV hydration should be administered depending on fluid loss.	Foot burn: Deep partial thickness burn on the dorsum of the foot with significant erythema and possible early skin breakdown. The intensity of the redness and tissue response suggests a deeper burn injury.	Foot Burn: Requires hospitalization due to depth and location. Elevated position, regular dressing changes with silver sulfadiazine or modern silver hydrofiber dressings. Will likely require surgical debridement and split-thickness skin grafting due to the depth of injury. Non-weight-bearing status initially. DVT prophylaxis is important due to immobility. Physical therapy involvement for maintaining range of motion and eventual gait training.	A foot with a 10 × 3 cm superficial partial-thickness burn on the lateral aspect. The burn has erythema and intact dermis. Mild edema and no visible infection.	Case 8 (10 × 3 cm superficial partial-thickness burn on foot): This superficial burn on the foot (<2% TBSA) can be managed ambulatorily. The wound should be cleansed with saline, treated with topical antibiotic ointment, and covered with a sterile dressing. Elevation of the affected limb and weight-bearing restrictions are recommended to reduce edema. Oral analgesics and tetanus prophylaxis are indicated. The patient should return in 24–48 h to assess for signs of infection or delayed healing. If the burn heals within 7–10 days, no further intervention is needed.
**Consensus surgeons answer**			Deep partial-thickness. Hospital admission due to depth and location. Non-weight-bearing initially. Dressing changes every 48 h with silver-based dressings. Pain management with oral or IV analgesia. Consider early debridement and STSG if healing is delayed beyond 2–3 weeks.				
9	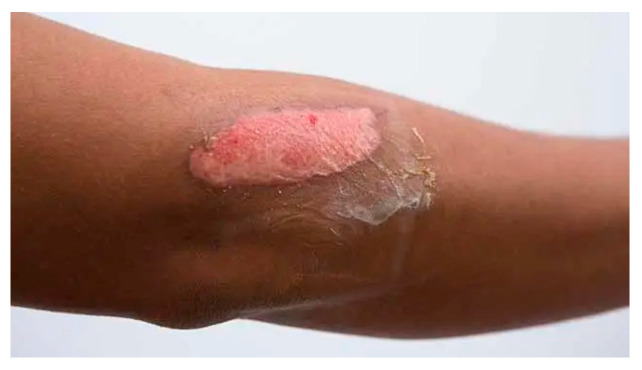	Intermediate second-degree burn on the posterior forearm. The image shows an oval-shaped burn on the posterior aspect of the forearm, with partial epidermal loss, exposing pinkish dermis. A thin layer of necrotic epidermis is peeling away, and the surrounding skin appears mildly edematous and erythematous. The wound is consistent with an intermediate second-degree burn.	The ninth case, an intermediate second-degree burn on the forearm, can be managed on an outpatient basis. Silicone-based foam or hydrocolloid dressings are appropriate to protect the wound and accelerate healing. Pain management with NSAIDs or paracetamol is sufficient. The patient should be monitored closely in follow-ups every 48–72 h to evaluate for signs of infection or delayed healing.	Arm lesion: Second-degree burn with clear blistering and surrounding erythema. The burn appears to be partial thickness with evident fluid accumulation under the epidermis.	Arm Blister: Can be managed as an outpatient. The blister should be left intact if clean to serve as a biological dressing. Apply non-adherent dressing like Mepitel with a secondary absorbent layer. Change dressings every 48–72 h. Oral analgesics for pain control. Arm elevation when resting. Follow up in 3–5 days to assess healing progress.	A 10 × 5 cm partial-thickness burn on a limb. The burn has red and white areas with blistering and a raised edge. Surrounding tissue is erythematous and edematous. No obvious signs of infection.	Case 9 (10 × 5 cm partial-thickness burn on limb): This burn (3–5% TBSA) requires hospital admission due to its size and depth. Initial management includes IV hydration (1–2 mL LR/kg/%TBSA), wound debridement, and application of topical antimicrobial agents (e.g., mafenide acetate). Pain control with IV opioids and tetanus prophylaxis are critical. Surgical consultation is advised for possible tangential excision and split-thickness skin grafting if healing is delayed beyond 10–14 days. The patient should be monitored for signs of systemic infection or compartment syndrome, particularly if the burn is circumferential.
**Consensus surgeons answer**			Superficial partial-thickness. Dressing changes, pain control with oral NSAIDs. Elevation to reduce edema. Monitor healing progress. Consider surgical debridement if no significant re-epithelialization by 10–14 days.				
10	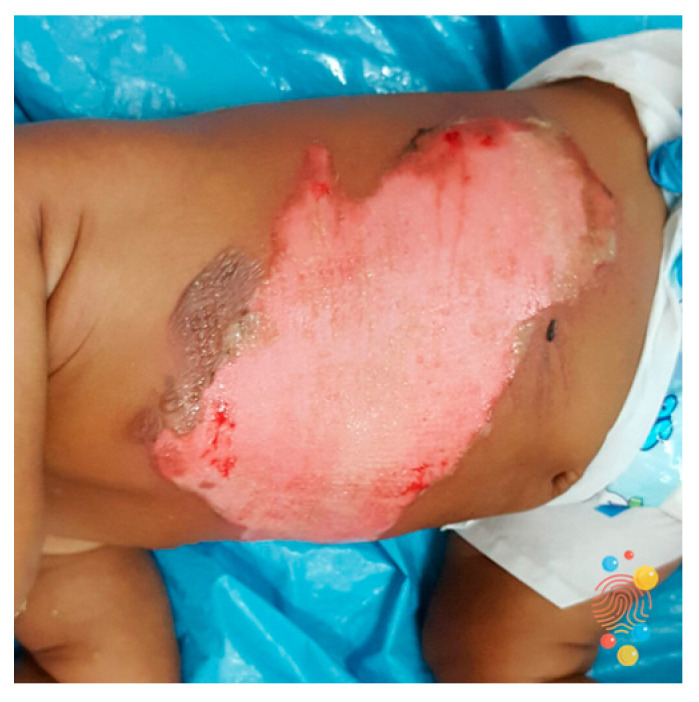	Extensive intermediate-to-deep second-degree burn on a pediatric patient’s abdomen. The image depicts a large burn on the lateral abdomen and flank of a child, with significant epidermal loss and exposed pink dermis. The wound has irregular borders, with some areas of necrosis and pinpoint hemorrhages, consistent with an intermediate-to-deep second-degree burn. The surrounding skin appears erythematous and edematous, with no visible signs of infection.	The final case, a pediatric patient with an extensive intermediate-to-deep second-degree burn on the abdomen and flank, is the most severe and requires hospital admission, potentially in an intensive care unit (ICU), depending on the total body surface area (TBSA) involved. Fluid resuscitation using IV hydration, guided by the Parkland formula, should be initiated if needed. The wound should be covered with silver-impregnated or silicone-based dressings, ensuring careful monitoring for infection. If there is no significant healing within 14–21 days, surgical debridement and skin grafting should be performed. Pain control may require opioids, and in severe cases, sedation might be necessary. Daily multidisciplinary evaluation is essential in this case.	Torso burn: Large partial thickness burn showing typical features of second-degree injury, including erythema, possible blistering, and clear margins. The burn appears to be of mixed depth with varying degrees of dermal involvement.	Torso Burn: Requires hospital admission due to size and location. Fluid resuscitation is necessary based on TBSA calculation. Regular vital sign monitoring and pain management with IV opioids initially. Daily dressing changes using silver-based products under sterile conditions. High-protein nutritional support is essential. May require surgical debridement and skin grafting if deep areas are present. Physiotherapy for chest care and mobility. A minimum of 7–10 days of admission likely needed.	A 20 × 15 cm partial-thickness burn on the back. The burn has red and white patches, with areas of blistering and fluid accumulation. The edges are indistinct, and the surface appears shiny due to exudate. Surrounding skin is erythematous. No visible signs of infection.	Case 10 (20 × 15 cm partial-thickness burn on back): This large burn (>20% TBSA) requires immediate hospitalization and ICU monitoring for fluid resuscitation (Parkland formula: 800 mL LR initially) and hemodynamic support. The wound should undergo emergent debridement and split-thickness skin grafting to prevent infection and sepsis. Analgesia (e.g., IV morphine) and tetanus prophylaxis are essential. The patient should be monitored for complications such as acute kidney injury, respiratory failure, or sepsis. Early mobilization and nutritional support (e.g., high-protein diet) are critical for recovery.
**Consensus surgeons answer**			Deep partial-thickness. Referral to pediatric burn center. Close monitoring and IV fluid resuscitation if TBSA is significant. Dressing changes with silver-based dressings. Consider surgical debridement and STSG if no healing within 14–21 days. Multidisciplinary follow-up is essential.				

Abbreviations: NSAIDs = non-steroidal anti-inflammatory drugs, IV = intravenous, TBSA = total body surface area, ICU = intensive care unit

**Table 2:** Likert scale evaluation of AI-generated burn case descriptions and management plans.

Case	ChatGPT Likert Case Description	ChatGPT Likert Management	Claude Likert Case Description	Claude Likert Management	Kimi Likert Case Description	Kimi Likert Management
1	5	5	5	5	4	4
2	5	4	4	4	4	4
3	5	5	5	4	4	4
4	5	5	4	5	4	4
5	5	5	4	4	4	4
6	4	4	5	5	4	4
7	5	5	4	5	4	4
8	4	4	5	4	4	4
9	5	5	4	5	4	4
10	5	5	5	5	4	4
Average	4.8	4.8	4.5	4.6	4	4

**Table 3:** Statistical analysis of Likert ratings for AI-generated burn case descriptions and management plans.

Statistic	ChatGPT Likert Case Description	ChatGPT Likert Management	Claude Likert Case Description	Claude Likert Management	Kimi Likert Case Description	Kimi Likert Management
Mean	4.80	4.70	4.50	4.60	4.00	4.00
Standard Deviation	0.42	0.48	0.53	0.52	0.00	0.00
Minimum	4	4	4	4	4	4
Maximum	5	5	5	5	4	4
Frequency of 4	2	3	5	4	10	10
Frequency of 5	8	7	5	6	0	0

**Table 4:** Statistical comparisons of AI model performance using Friedman and Wilcoxon tests.

Comparison	Value
Friedman Test (Description) Chi-square (df = 2)	6.20
Friedman Test (Description) *p*-value	0.045
Wilcoxon ChatGPT vs. Kimi (Description) *p*-value	0.040
Wilcoxon ChatGPT vs. Claude (Description) *p*-value	0.070
Wilcoxon Claude vs. Kimi (Description) *p*-value	0.500
Friedman Test (Management) Chi-square (df = 2)	4.80
Friedman Test (Management) *p*-value	0.090
Wilcoxon ChatGPT vs. Kimi (Management) *p*-value	0.120
Wilcoxon ChatGPT vs. Claude (Management) *p*-value	0.150
Wilcoxon Claude vs. Kimi (Management) *p*-value	0.300
Correlation (Spearman) Desc vs. Mng (ChatGPT)	0.72
Correlation (Spearman) Desc vs. Mng (Claude)	0.65
Correlation (Spearman) Desc vs. Mng (Kimi)	0.10

## Data Availability

The authors confirm that the data supporting this study’s findings are available within the article.

## Abbreviations

The following abbreviations are used in this manuscript:

AI	Artificial intelligence
CNNs	Convolutional neural networks
NLP	Natural language processing
NSAIDs	Non-steroidal anti-inflammatory drugs
IV	Intravenous
TBSA	Total body surface area
ICU	Intensive care unit
EV	Endovenous
FANS	Non-steroidal anti-inflammatory drugs (European term)
LASCA	Laser Speckle Contrast Analysis
